# Islet-Like Cell Aggregates Generated from Human Adipose Tissue Derived Stem Cells Ameliorate Experimental Diabetes in Mice

**DOI:** 10.1371/journal.pone.0020615

**Published:** 2011-06-07

**Authors:** Vikash Chandra, Swetha G, Sudhakar Muthyala, Amit K. Jaiswal, Jayesh R. Bellare, Prabha D. Nair, Ramesh R. Bhonde

**Affiliations:** 1 Tissue Engineering and Banking Laboratory, National Centre for Cell Science, Ganeshkhind, Pune, Maharashtra, India; 2 Biomedical Technology Wing, Sree Chitra Tirunal Institute for Medical Sciences and Technology, Thiruvananthapuram, Kerala, India; 3 Department of Chemical Engineering, Indian Institute of Technology Bombay, Mumbai, Maharashtra, India; University of Bremen, Germany

## Abstract

**Background:**

Type 1 Diabetes Mellitus is caused by auto immune destruction of insulin producing beta cells in the pancreas. Currently available treatments include transplantation of isolated islets from donor pancreas to the patient. However, this method is limited by inadequate means of immuno-suppression to prevent islet rejection and importantly, limited supply of islets for transplantation. Autologous adult stem cells are now considered for cell replacement therapy in diabetes as it has the potential to generate neo-islets which are genetically part of the treated individual. Adopting methods of islet encapsulation in immuno-isolatory devices would eliminate the need for immuno-suppressants.

**Methodology/Principal Findings:**

In the present study we explore the potential of human adipose tissue derived adult stem cells (h-ASCs) to differentiate into functional islet like cell aggregates (ICAs). Our stage specific differentiation protocol permit the conversion of mesodermic h-ASCs to definitive endoderm (Hnf3β, TCF2 and Sox17) and to PDX1, Ngn3, NeuroD, Pax4 positive pancreatic endoderm which further matures in vitro to secrete insulin. These ICAs are shown to produce human C-peptide in a glucose dependent manner exhibiting in-vitro functionality. Transplantation of mature ICAs, packed in immuno-isolatory biocompatible capsules to STZ induced diabetic mice restored near normoglycemia within 3–4 weeks. The detection of human C-peptide, 1155±165 pM in blood serum of experimental mice demonstrate the efficacy of our differentiation approach.

**Conclusions:**

h-ASC is an ideal population of personal stem cells for cell replacement therapy, given that they are abundant, easily available and autologous in origin. Our findings present evidence that h-ASCs could be induced to differentiate into physiologically competent functional islet like cell aggregates, which may provide as a source of alternative islets for cell replacement therapy in type 1 diabetes.

## Introduction

Type 1 diabetes is characterized by the autoimmune destruction of β cells, resulting in life-long dependency on insulin injection that often results in complications of hypo- or hyperglycemia. Although Edmonton protocol for islet transplantation is the most preferred therapy available for type 1 diabetes, a major obstacle with this therapy is the limited supply of cadaveric donor islets in retention to the high demand of eligible patients and the need for lifetime immunosuppressant [1]–[4]. Recent studies have demonstrated that Embryonic stem cells (ESCs) [5]–[7], Induced pluripotent stem cells (IPSs) [8], [9], and adult stem cells like bone marrow (BM) [10], pancreas [11], [12], liver [13], umbilical cord blood [14], Wharton's jelly [15], placenta [16], could be differentiate into insulin producing cells. ESCs have tremendous pluripotency, however, ethical/legal issues and risks of teratoma formation limit its use in translational medicine. In this scenario, Adipose-derived adult stem cells (ASCs) appear to be an ideal population of stem cells for practical cell replacement therapy, given that they are abundant, autologous tissue and ease in availability [17]. Even lean adult men and women have at least 3.0–4.5 kg of adipose tissue, and in individuals with severe obesity, adipose tissue can constitute up to 45 kg or more of body weight [18], sufficient quantities of ASCs can be harvested and cultured from a minimum of 1.0 g of fat. Moreover adipose tissue is a remarkable organ that play important role in glucose homeostasis and hormone production (adipokine) [19]. Some of the adipokines like leptin and adiponectin have direct impact on glucose homeostasis and fatty acid oxidation [20].

Findings by Timper et al [21] and Lee et al [22] with human ASCs and our earlier experience with murine ASCs [23] indicate that h-ASCs are the ideal candidates for cell replacement therapy in diabetes.

In the present study we explore the potential of h-ASCs to differentiate into functional islet like cellular aggregates (ICAs) with stage specific differentiation conditions. The differentiated ICAs are packed in immunoisolation capsules for transplantation studies. These ICAs when transplanted into STZ induced diabetic mice can restore near normoglycemia within 3–4 weeks. The detection of human specific C-peptide in transplanted mice serum further strength our differentiation approach. Our studies thus raise the possibility that patient own adipose tissue can serve as a source of ASCs to differentiate into functional autologous ICAs for cell replacement therapy in type 1 diabetes.

## Results

**Figure 1:**
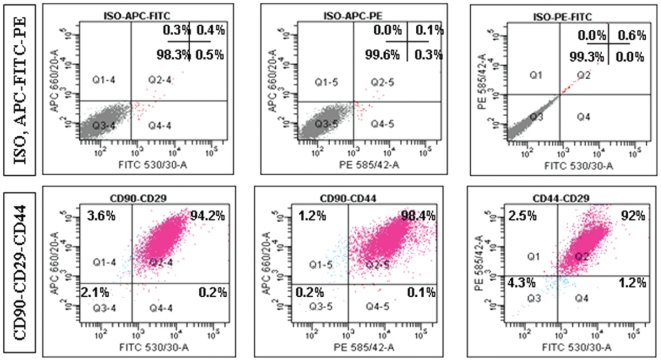
Uniform co-expression of CD90-CD29-CD44 in clonally expanded h-ASCs. Flow cytometry based characterization of the clonal population of h-ASCs. Representative profile of clonally expanded h-ASCs (n = 4); cells are triple positive for CD90, CD44 and CD29 (>90%) antigens. Percentage of positivity indicated in top right corner. Three different color conjugated antibodies were used, CD90-APC, CD44-PE and CD29-FITC. Isotype controls were used for all antibody combinations.

Human adipose tissue derived adult stem cells (h-ASCs) were isolated from the resected fat tissue samples (n = 6, female donor of age group 25–50 years) as earlier reported [17], [21]. The initial culture of stromal vascular fraction resulted in the growth of plastic adherent cell population with typical mesenchymal morphology. Although freshly isolated h-ASCs showed heterogeneous phenotype in culture, single fibroblastoid cell populations were clonally expanded which exhibited homogenous morphology. Four clones of h-ASCs were evaluated for all the differentiation studies and all experiments were carried out using h-ASCs of passages 4–8. The homogeneity of the cloned population was confirmed by the triple stained FACS analysis of h-ASCs at passage-4 which showed uniform co-expression of CD90, CD44 and CD29 surface markers (Figure 1).

**Figure 2:**
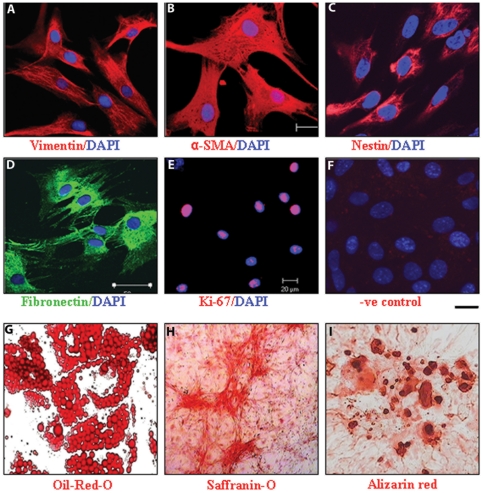
In vitro characterization of clonally expanded h-ASCs for mesenchymal markers. Representative immunostaining of cytoskeletal protein vimentin (A), alpha smooth muscle actin (B), nestin (C), fibronectin (D) and cell proliferation marker Ki-67 (E) with immunofluoresence staining. Negative control staining with fluorescence conjugated secondary antibodies shown (F). Cell nuclei were stained with DAPI. Scale bar, 20 µm. h-ASCs exhibited competence to differentiate into adipogenic, osteogenic and chondrogenic lineages in-vitro upon specific induction as confirmed by Oil red O (G), Saffranin-O (H) and Alizarin-red (I) staining respectively.

Surface phenotype characterization of h-ASCs with flow cytometry showed that, unlike murine ASCs, h-ASCs showed high expression of CD13, CD59, CD105 (Figure S1). h-ASCs showed expression of cytoskeletal proteins vimentin, alpha-smooth muscle actin, nestin and extracellular matrix protein fibronectin (Figure 2, A–D). Cells were highly mitotic in culture and expressed proliferation marker Ki-67 (Figure 2E). h-ASCs exhibited in-vitro competence to differentiate into adipogenic, chondrogenic and osteogenic lineages upon specific induction as confirmed by Oil red O (Figure 2G), Saffranin-O (Figure 2H) and Alizarin-red staining respectively (Figure 2I).

### Differentiation of h-ASCs to definitive endoderm ICAs

**Figure 3:**
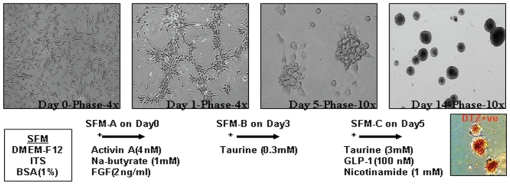
Differentiation strategy for h-ASCs derived ICAs, day 14 ICAs stained positive with islet specific DTZ staining. h-ASCs in monolayer when exposed to SFM A undergo cell clustering and aggregation. Subsequent exposure and incubation of h-ASCs in SFM B on day 3 and SFM C on day 5 results in the formation of mature Islet like cell aggregates (ICAs). By day14, the ICAs become fully differentiated and mature and stain positive for DTZ. Abbreviations: SFM, Serum free media; ITS, Insulin-transferrin-selenium; BSA, Bovine serum albumin, GLP- Glucagon Like Peptide, DTZ, dithizone.

**Figure 4:**
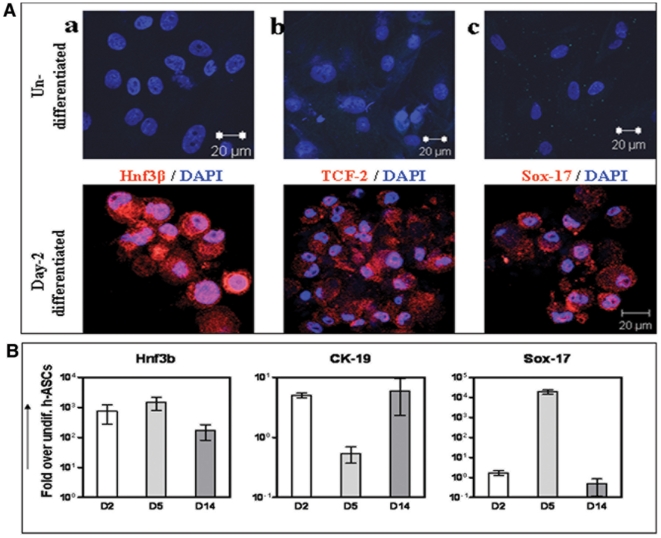
Characterization of h-ASCs derived ICAs for endoderm markers. Immuno-staining of ICAs exposed to SFM A on day-2 for the expression of definitive endoderm markers Foxa2 (HNF3B) (A-a), TCF-2 (A-b) and Sox-17 (A-c) as compared to undifferentiated h-ASCs (top panel of Figure 4). Nuclei stained with DAPI. Scales bar = 20 µm. qRT-PCR data on the expression of definitive endoderm markers Foxa2, CK-19 and Sox-17 during specific stages of differentiation (day 2, day 5 and day 14) ICAs and compared with undifferentiated h-ASCs. All mRNA expression levels were normalized to the house keeping gene GAPDH expression, data presented here as fold increase of mean±S.E.M over h-ASCs undifferentiated cells (B). Abbreviations: DAPI, (4′, 6-diamidoino-2-phenylindole); CK-19, cytokeratin-19; qRT PCR, quantitative reverse transcription polymerase chain reaction; GAPDH, glyceraldehyde-3-phosphate-dehydrogenase.

h-ASCs were grown in low adherence culture dishes and exposed to serum free media (SFM-A) for 2 days. By 24–48 hrs, cells migrated and clustered to become islet like cells aggregates (ICAs) (Figure 3). By day 2, the cells started expressing endoderm markers like HNF-3 beta, TCF-2 and Sox-17 as compared to undifferentiated h-ASCs (Figure 4A a–c). qRT-PCR analysis of day2-ICAs showed increased transcript abundance of the endoderm markers HNF-3 beta/FoxA2 (10^3^ fold), CK-19 (10 fold) and Sox-17 (2 fold) as compared to undifferentiated h-ASCs (Figure 4B) The highest mRNA expression levels of Sox17, a definitive endoderm marker was observed by day 5.

### Pancreatic Endoderm differentiation of ICAs derived from h-ASCs

**Figure 5:**
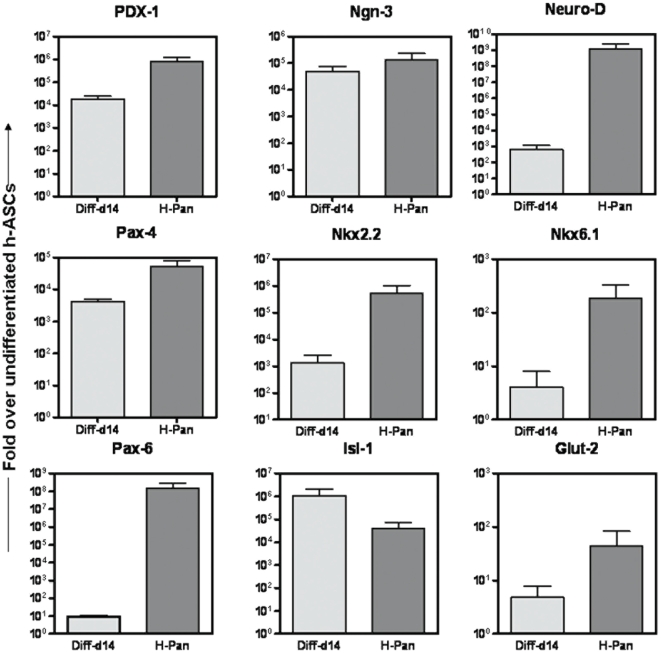
Expression profile of pancreatic endoderm genes during the course of differentiation. h-ASCs when differentiated to mature ICAs expressed various genes involved in pancreas development. SYBR-Green based qRT PCR was performed for the pancreatic progenitor specific transcription factors like PDX1, Ngn3, NeuroD, Pax4, Nkx2.2, Nkx6.1, Pax6, Isl1 and glucose transporter Glut-2 at day14 (Diff-d14) of differentiation. Gene transcripts of ICAs are compared with human fetal pancreas (12 week) (H-Pan). Relative levels of gene expression were normalized to the GAPDH mRNA level. Transcript abundance is shown here as fold increase of mean±S.E.M. for two experiments and represented as fold over undifferentiated h-ASCs. Abbreviations: Diff-d14: Differentiated ICAs on day 14 and H-Pan: Human fetal pancreas- 12 week).

In the next stage, to achieve pancreatic endoderm differentiation day2-ICAs were exposed to SFM supplemented with taurine (SFM-B), a non-essential amino acid involved in the development of pancreatic β-cells [24], [25]. After 2 days of incubation in SFM-B, in the final stage of differentiation, ICAs were exposed to differentiation media supplemented with β-cell maturation factors like GLP-1 and nicotinamide (SFM-C) and maintained for additional 7–8 days. A significant up regulation in the expression of pancreatic endoderm markers as well as key transcription factors involved in pancreas development like PDX-1, Ngn3, NeuroD, Pax-4, Nkx2.2, Nkx6.1, Pax-6, Isl-1 and glucose transporter Glut-2 were observed in d14- ICAs over undifferentiated h-ASCs (Figure 5).

Transcript levels of PDX-1, Ngn-3, Pax-4 and Isl-1 in d14 ICAs were comparable to that of 12 week old human fetal pancreas. By day-14 of differentiation the ICAs stained positive for DTZ, a zinc-chelating agent known to selectively stain pancreatic beta cells (Figure 3).

**Figure 6:**
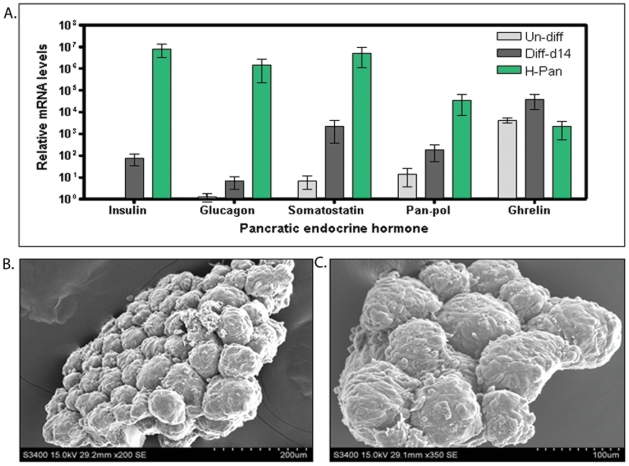
Expression of pancreatic endocrine hormones transcript during the course of differentiation, surface topography of day 14 ICAS with scanning electron microscopy. qRT PCR based transcript abundance of pancreatic hormones (Insulin, Glucagon, Somatostatin, Pan-pol and Ghrelin) in undifferentiated and day 14 differentiated h-ASCs. Transcript abundance of these genes was also compared to that of human fetal pancreas 12 week (H-Pan). Relative levels of gene expression were normalized to the GAPDH mRNA level and presented here as fold increase of mean±S.E.M over detectable (Ct of 39) (A). (White bar: Undifferentiated h-ASCs, Grey bar: Differentiated ICAs on day 14 and Green bar: Human fetal pancreas- 12 week). Scanning electron microscopy (SEM) of day 14 ICAs show typical surface topography similar to that of pancreatic islets. Scale bar = 200 µm (B) and 100 µm (C). Abbreviations: qRT PCR, quantitative reverse transcription polymerase chain reaction; cDNA, complementary DNA; pan pol, pancreatic polypeptide; GAPDH, glyceraldehyde-3-phosphate-dehydrogenase.

The mature day-14 ICAs showed enhanced transcript levels of insulin, glucagon, somatostatin, pan-polypeptide and ghrelin as compared to undifferentiated h-ASCs. The transcript abundance of pancreatic hormones in mature ICAs was lower than that of human fetal pancreas (12 week). Undifferentiated h-ASCs consistently exhibited transcripts of ghrelin (Figure 6A). Scanning electron microscopy based ultra structural analysis showed typical islet like surface topography and organization in day 14 ICAs (Figure 6B).

**Figure 7:**
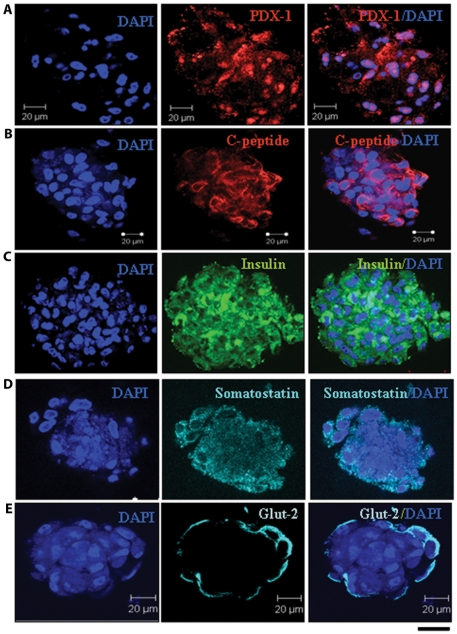
Confocal immunocytochemistry of day14 ICAs shows the expression of pancreatic endoderm markers. Confocal optical sections of ICAs stained for PDX-1(a), C-peptide (b), Insulin (c), Somatostatin (d), and Glut 2 (e). Nuclei are stained by DAPI (4′, 6-diamidoino-2-phenylindole). (Scale bar = 20 µm).

These results were further confirmed by immuno-staining of matured day-14 ICAs for islet specific transcription factors and hormones. Matured day-14 ICAs exhibited abundant expression of Pdx-1 (Figure 7A), C-peptide (Figure 7B), insulin (Figure 7C), somatostatin (Figure 7D) and glucose transporter2 (Figure 7E). Similar to our earlier observation with murine ASCs derived ICAs (23), the Day 14 human ICAs also showed co-expression of insulin and somatostatin (Figure S2A). The mature ICAs also showed proliferative potential depicted by Ki-67 expression (Figure S2B). The gene profiling studies for adipose tissue-specific markers during ICA differentiation showed that the transcript level of leptin and adiponectin in mature ICAs were comparable to that of h-ASCs (Figure S3A). Immunostaining of h-ASCs and d14-ICAs showed low levels of leptin expression (Figure S3B).

### Static stimulation and total C-peptide content of mature ICAs

**Figure 8:**
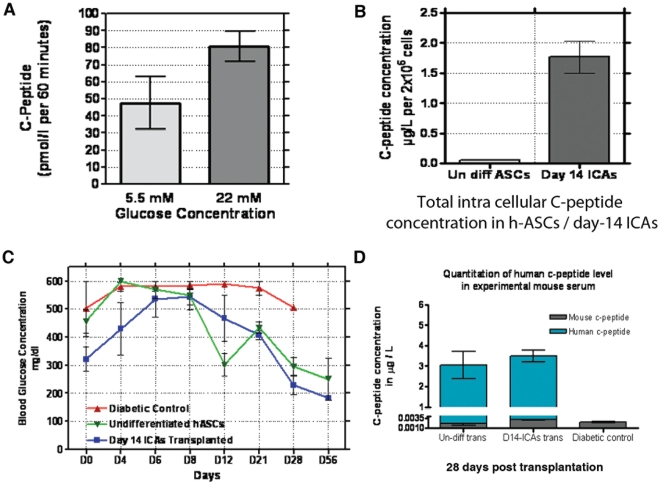
In-vitro and in-vivo functional characterization of ICAs. Static stimulation assay for day14 ICAs. ICAs were sequentially treated with low glucose (5.5 mM) and high glucose (22 mM) concentration in triplicate for one hour. Cell supernatants were collected and analyzed for c-peptide release by EIA assay (*p* values≤0.05) (n = 3) (A). Total intracellular c-peptide content of day 14 ICAs (*p*≤0.05) was measure in acid-ethanol and compared equal number of undifferentiated h-ASCs (*p*≤0.05), (n = 3) (B). In-vivo characterization of h-ASCs derived ICAs. Transplantation of day-14 ICAs in STZ induced diabetic Swiss albino mice (n = 3+3) showed hypoglycemic effect within two weeks. Day-14 ICAs (1000–1200) were encapsulated in calcium-alginate and packed into biocompatible capsules (made of PU-PVP-IPN) and transplanted intraperitoneally to diabetic mice. Control group of animals included sham control of diabetic mice transplanted with empty capsule (n = 3) and diabetic mice transplanted with encapsulated undifferentiated h-ASCs (n = 3+3). Overnight fasting blood glucose levels of mice in each experimental group for three week time period is represented (C). Quantification of human C-peptide in blood serum of mice transplanted with ICAs and diabetic control mice (D). Serum samples of experimental mice were collected by retro orbital bleeding on 28^th^ day of transplantation, for the detection of human c-peptide/mouse c-peptide in blood with ultrasensitive human c-peptide/mouse c-peptide kit were used. Abbreviations: ICAs, Islet like cell aggregates; STZ, streptozotocin; IP-GTT, intra-peritoneal glucose tolerance test; DAPI, (4′, 6-diamidoino-2-phenylindole).

In this study, day-14 h-ASCs derived ICAs were first incubated with 5.5 mM glucose for 1 h to measure the basal level of c-peptide release which is a direct indicator of the amount of de novo insulin released. The same ICAs were subsequently incubated for additional 1 h in 22 mM glucose to measure glucose-stimulated C-peptide release (n = 3). Total C-peptide content of day 14 ICAs measured up to 47.68±15.20 pM per 60 min when exposed to 5.5 mM glucose (*p* = 0.070). When stimulated with 22 mM glucose, the total C-peptide content of ICAs increased to 80.61±8.92 pM per 60 minute (*p* = 0.05) confirming their in vitro functionality and ability to respond to glucose (Figure 8A). Total intracellular C-peptide content of day 14 differentiated ICAs was calculated and value was compared with undifferentiated h-ASCs (equal number of starting cells (2×10^6^ cells) were taken to compare the two values). It was observe that h-ASC derived day 14 ICAs contained up to 1.76±0.45 µg/l (582.56±148.95 pM) (*p* = 0.02) as compared to 0.051±0.006 µg/l (*p* = 0.005) of C-peptide in undifferentiated h-ASCs (n = 3) (Figure 8B). However in day-14 ICAs cell numbers are potentially not equal and therefore this makes direct comparison of C-peptide content difficult.

### Mature ICAs show hypoglycemic effect in experimental diabetic mice

The physiological competence of mature day-14 ICAs to maintain glucose homeostasis in vivo was evaluated by transplantation of ICAs in STZ induced diabetic mice. ICAs (1000–1200) encapsulated in calcium-alginate were packed into biocompatible capsules of PU-PVP-IPN [26] and transplanted into the peritoneal cavity of diabetic mice (n = 6). By 2–3 weeks, mice transplanted with mature ICAs showed reduction in blood glucose levels. The mice maintained lowering of blood glucose (180–190 mg/dl) upto eight week of follow up. However, it was observed that normoglycemia was not restored in these mice. Transplantation of undifferentiated h-ASCs (n = 6) to STZ-induced diabetic mice also showed lowering of blood glucose (250 mg/dl) which was maintained but failed to restore normoglycemia within the stipulated time (Figure 8C). To determine whether the ICAs could regulate blood glucose levels independently of the endogenous pancreatic β-cells, we measured level of human as well as mouse C-peptide in blood serum of all experimental mice. We measured up to 1155±165 pM (3.49±0.50 µg/l) serum level of human C-peptide in diabetic mice transplanted with day 14 ICAs on day 28 post transplantation. The level of human C-peptide in mice transplanted with undifferentiated h-ASCs (2×10^6^), 28^th^ days post transplantation measured to 1009±383 pM (3.05±1.16 µg/l) The mouse C-peptide concentration was found to be very low to undetectable (∼0.002 µg/l) in control diabetic mice (Figure 8D). ICAs retrieved from the transplanted mice after 4 weeks showed good cellular aggregation and viability (data not shown). The ICAs and undifferentiated h-ASCs retrieved from the transplanted mice showed insulin and C-peptide expression (Figure S4A and B).

## Discussion

In this study we assessed the potential of h-ASCs to produce functional pancreatic hormone producing islet like cell aggregates from resected human fat samples. Our isolation protocol for Stromal vascular fractions consistently yielded multipotent mesenchymal cells expressing CD29+/CD44+/CD90+ surface antigen profile. These cells demonstrated high competence to differentiate to adipogenic, osteogenic and chondrogenic lineages. h-ASCs isolated in culture differed in its expression of surface antigens from murine ASCs isolated by similar procedure. h-ASCs distinctly showed high expression of CD90 and low expression level of Sca-1 as against m-ASCs [23]. The surface antigen profile differed from human BM derived mesenchymal cells as well indicating that despite the similarities in morphology, mesenchymal cells isolated from different sites/tissue of origin possess distinct molecular signatures and identities. In accordance with earlier reports, we observed a stringent dependence of h-ASCs on FGF for proliferation and survival in vitro [27]. Subsequently all media and differentiation cocktails were supplemented with 2 ng/ml FGF.

To achieve differentiation to pancreatic lineage, specific step-wise conditions were formulated as per earlier reported protocol [23]. We achieved pancreatic endoderm differentiation of the mesodermic h-ASCs with activin A, sodium butyrate, 2-mercaptoethanol, ITS and 2 ng/ml FGF (SFM-A). We found that addition of FGF stimulate the initial process of cell aggregation and cluster formation, an important step in the development and differentiation of pancreas via FGF-receptor on h-ASCs [28]. Definitive endoderm differentiation was identified by the expression of Hnf3β/Foxa2 in (∼80%) [29], TCF-2 [30] and Sox-17 [31] in 50%–60% cells in day 2 differentiated cells. Increased mRNA expression of Hnf3β, CK-19 and Sox-17 in comparison to undifferentiated h-ASCs, further confirmed their definitive endoderm differentiation. Sox-17 and Hnf3β expressions were highest during day-5 of differentiation. The expression of these genes occurs in a similar pattern as observed during pancreatic development. The low cell attachment culture dish and initial high plating density (2×10^6^ cells generated around 400–600 ICAs) proved to be highly efficient in helping cells to form 3D aggregates similar to our earlier observations with murine ASCs. By 24–36 h of initial plating, h-ASCs formed clusters (ICAs) with compact cellular aggregation.

Previous studies have shown that supplementation of taurine, a non essential amino acid modulate glucose homeostasis and islet function [24],[25]. The taurine supplemented with nicotinamide and GLP-1 in the differentiation cocktail resulted in the up regulation of a number of genes (PDX1, Ngn3, NeuroD, Pax4, Nkx2.2, Nkx6.1, Pax-6, Isl-1 and pancreas specific glucose transporter Glut-2) involved in pancreatic beta cells development cascade. Additionally, detectable level of transcripts of two pancreatic progenitor specific transcription factors Nkx2.2/Nkx6.1 and Isl-1 in undifferentiated h-ASCs make it more suitable candidate stem cells to differentiate into pancreatic lineage. Nkx2.2 and its down stream factor Nkx6.1 participate in the major pathway of β cell formation in the pancreas and knockout mice of these factors develop with reduced number of mature β cells [32].

Mature ICAs showed transcript abundance and expression of pancreatic endocrine hormones. We observed that cells within the differentiated ICAs co-express insulin and somatostatin which is in concordance with earlier reports on newly differentiated midgestational human fetal pancreas cells which co express multiple hormones [33], [34]. Glucose static stimulation index of mature d 14 ICAs measured upto 1.69 for C-peptide. Total intracellular human C-peptide content in day 14 ICAs was 1.76±0.45 µg/l. As our differentiation cocktail includes ITS, to rule out any possibilities that insulin might be absorbed by cells during in vitro culture [35], [36], we always calculated the level of human c-peptide for de novo synthesis of insulin. Previous reports on the differentiation potential of human ASCs by Timper et al and Lee et al [21], [22] although novel, did not demonstrate in vitro/invivo functionality of the differentiated cells. To the best of our knowledge this is the first study demonstrating in vitro/in vivo functionality of ICAs differentiated from h-ASCs.

STZ induced mice were considered diabetic when the blood glucose levels were higher than 200 mg/dl. For in vivo transplantation studies we used PU-PVP-IPN biocompatible capsules as an immuno-isolatory devise. Our initial studies have shown that a minimum of 1000–1200 ICAs/capsule/mice is required to achieve significant hypoglycemia in STZ induced diabetic mice. The capsule exhibited biocompatibility and did not evoke any immune rejection in the implanted mice. The porosity of the capsule is such that it easily allows nutrient exchange between the packed ICAs and the peritoneal cavity. By three weeks, all the mice transplanted with ICAs, showed lowering of blood glucose levels and maintained the status for two months of tracking. However, transplanted ICAs could not bring down blood glucose to normal physiological levels. This may be circumvented by optimizing the number of ICAs to be transplanted to experimental mice. To determine whether the h-ASCs derived ICAs could regulate blood glucose levels independently of the mice endogenous pancreatic β cells; we have estimated human C-peptide level in serum of experimental mice. Random serum levels of human C-peptide after four weeks of transplantation was measured up to 1155±165 pM (3.49±0.50 µg/l) (n = 3), is comparable with the earlier reports with human embryonic stem cells [37] where they found serum levels of human C-peptide up to 852±263 pM fasting and 2361±565 pM in glucose stimulated. It was interesting to observe that transplantation of undifferentiated h-ASCs to STZ induced diabetic mice showed moderate lowering of blood glucose levels, which also reflected in the serum levels of human C-peptide1009±383 pM (3.05±1.16 µg/l). This shows that the autocrine and paracrine factors of regenerating pancreas and hyperglycemic local diabetic micro-environment of mice may contribute to a small extend, the differentiation of ASCs as earlier reported for human bone marrow MSCs [38]. This observation calls for detail studies to explore the potential of h-ASCs to differentiate into pancreatic lineage and regulate glucose homeostasis with minimum manipulation. We also checked the pattern of some of adipokines (leptin and adiponectin) during the course of differentiation. It was found that the after initial down regulation of these genes they reappear at the end of differentiation. Leptin and adiponectin are considered to be good adipokines that play important role in glucose homeostasis and fatty acid oxidation. The role of adipokines in glucose metabolism is a highly studied subject. Earlier reports show that in-vitro chronic exposure of human islets to leptin induced IL-1beta, leading to impair beta cell function [39]. However recent studies demonstrate that Leptin monotherapy improves several of the metabolic imbalances caused by insulin deficient type 1 diabetes in rodents by CNS dependent mechanism [40]. It would be interesting to find out whether these adipokines play any regulatory role in glucose homeostasis in h-ASC derived ICAs. Scanning electron microscopy based ultra-structural comparison of these 3D ICAs revealed that their surface topography and arrangement between cells is very similar to that of normal pancreatic islets [41]. All the four clones used in this study exhibited equal frequency of in vitro differentiation and maturation to ICAs. The inherited expression of Isl-1/Pax-6/Nkx6.1/Somatostatin transcripts in h-ASCs, its ease in availability and autologous origin, make it an eminent candidate for cell replacement therapy in diabetes. To the best of our knowledge this is the first detailed report demonstrating the differentiation potential of human ASCs to glucose responsive pancreatic hormone expressing islet like cellular 3D aggregates. We anticipate that our work would add on to the ongoing research to find alternative sources of islets for cell replacement therapy in type 1 diabetes.

### Conclusion

Our findings present evidence that h-ASCs could be induced to differentiate into physiologically functional islet like cell aggregates, which may provide a source of alternative islets for cell replacement therapy in type 1 diabetes.

## Materials and Methods

### ASCs Cell Isolation and Expansion

Resected fat tissue samples were obtained during different abdominal surgeries from female donors (20 to 40 years old). Collection of the sample was approved by the institutional review board (IEC-Institutional ethical committee, NCCS, National Center for Cell Science, Pune) and written consent was obtained from all donors. Adipose tissue samples (n = 6) were washed and digested in PBS supplemented with 2% bovine serum albumin (BSA) and 0.2% collagenase-type-II (Sigma-Aldrich, St. Louis, http://www.sigmaaldrich.com) pre-warmed to 37°C for 45 minutes. The cells were centrifuged (400 g for 4 minutes) at room temperature (RT) in 1–2 ml sterile PBS containing 2% (v/v) fetal bovine serum (FBS) (Sigma-Aldrich). Supernatant containing mature adipocytes were discarded. The pellet containing stromal vascular fraction was resuspended in expansion medium.

#### Expansion medium

Dulbecco's modified eagle's medium [DMEM]/Ham's F-12 (GIBCO) with 10% FBS, antibiotics and supplemented with 2 ng/ml Fibroblast Growth Factor (FGF, Sigma-Aldrich #F0291). . The cells were plated at density of 2×10^5^/cm^2^ into T25 culture flasks.

Serial dilution method was used to generate single-cell clonogenic culture. Briefly, 10^4^ isolated human ASCs (h-ASCs) were suspended in 1 ml culture medium. Repeated serial dilution was carried out to achieve a final dilution of 100 cells in 10 ml medium. For single-cell culture, 100 µl of the diluted cell suspension was transferred into each well of a 96-well plate containing 100 µl of culture medium.

### In Vitro Differentiation of mE-ASCs to ICAs

Differentiation was carried out in three stages as reported earlier [23]. Cells were counted for initial seeding density and 1×10^6^ cells/cm^2^ were resuspended in SFM-A and plated to ultra-low attachment tissue culture plates (Corning, Fisher scientific) or small glass petri plate (8×10^6^ Cells in 2″ glass petri plate makes ∼800–1200 ICAs). SFM-A contained DMEM/F12 (1∶1) (GIBCO) with 17.5 mM glucose, 1% BSA Cohn fraction V, fatty acid free (#A8806, Sigma-Aldrich), 1× Insulin-transferrin-selenium (ITS, 5 mg/L insulin+5 mg/L transferrin+5 mg/L selenium), 4 nM activin A, 1 mM sodium butyrate, 50 µM 2-mercaptoethanol and 2 ng/ml Fibroblast Growth Factor. The cells were cultured in this media for 2 days. On 3^rd^ day media was changed to SFM-B that contained DMEM/F12 (1∶1) with 17.5 mM glucose, 1% BSA, ITS and 0.3 mM Taurine. The cell aggregates were cultured in this medium for another 2 days and shifted to SFM-C on 5^th^ day. SFM-C contained DMEM/F12 (1∶1) with 17.5 mM glucose, 1.5% BSA, ITS, 3 mM Taurine, 100 nM Glucagon-like peptide 1 (GLP-1) (amide fragment 7–36, Sigma Aldrich), 1 mM nicotinamide and 1× non-essential amino acids (NEAA). The cell aggregates were fed with fresh SFM-C medium every 2 days for another 12–14 days. All chemicals were purchased from Sigma Aldrich unless otherwise indicated.

### Quantitative Real-Time Polymerase Chain Reaction

Tissue/Cells samples were frozen in Trizol (Invitrogen, Carlsbad, CA). Total RNA was isolated from duplicate and triplicate samples as per the manufacturers' instructions, measured on ND-100 spectrophotometer (Nanodrop Techno lies, Wilmington, DE) and 2 µg of RNA was used for cDNA synthesis per 20 µl reaction. cDNA was amplified using Reverse Transcription System Kit (ImPromII Reverse-transcription-system(#A3800), Promega-Corporation, Madison, USA). The primer-sequences used for quantitative real-time polymerase chain reaction (qRT-PCR) are summarized in supplementary Table S1. qRT-PCR was performed in duplicate and triplicate of total 25 µl reaction mixture containing 1× *Power* SYBR-Green Master-mix (Applied-Biosystems, http://www.appliedbiosystems.com), 600–750 nM each forward and reverse primers using 1/20^th^ of the cDNA preparation. PCR amplification was carried out using Applied-Biosystem7300 Real-Time PCR Sequence detection System (SDS-v1 3.1; Applied-Biosystems) (program: 2 minutes at 50°C, 10 minutes at 95°C, and 40 cycles of 15 seconds at 95°C and 1 minute at 60°C). All qRT-PCR results were normalized to GAPDH carried out in parallel in duplicate to correct differences in RNA input. For estimation of fold changes by qRT-PCR when the initial transcript levels were undetectable, the initial Ct value was assigned to be 39, which would lead to a possible underestimation of the actual fold change. The qRT-PCR values are mean± S.E.M.

### Immunophenotyping (Flow-Cytometric analysis)

Flow-cytometry was performed on a BD-FACS-Canto™ Flow-cytometer (www.bdbiosciences.com) and analysis was done with BD-FACS Diva softwarev5.0. Cells were harvested in 0.25% trypsin/EDTA, washed in Flow-cytometry buffer (FCB) (1× PBS, 0.5%FBS, 0.5%BSA, 0.05%sodium-azide). Cells were stained with the PE or FITC conjugated antibodies. ICAs were dissociated with 0.25% trypsin-EDTA (Sigma-Aldrich) at 37°C for 10–12 minutes. The sources of antibodies and dilutions used are summarized in supplementary Table S1.

### Immuno-cytochemistry (Confocal-microscopy)

Cultured cells or ICAs were fixed with freshly prepared 4% Para-formaldehyde (PFA) for 10 minutes at RT and permeabilized with either 0.1% Triton X-100 for 3–5 minutes on ice or with 50% chilled methanol for 20 minute on ice. Blocking done with blocking buffer (PBS, 0.5% FBS and 0.5% BSA) for 30 min at RT. Primary antibodies were incubated overnight at 4°C, washed with PBS and then incubated with the secondary antibodies at RT for 1 h. Slides were washed in PBS and mounted with Vectashield (Vector-Laboratories, CA, http://www.vectorslab.com). DAPI (4′, 6-diamidoino-2-phenylindole) (Invitrogen) was used to visualize nuclei. The sources of antibodies and dilutions used are summarized in supplementary table S1. Confocal images were captured using a Zeiss-LSM 510 laser scanning microscope using a 63×/1.3 oil objective with optical slices. ∼1–2 µm. All results are representative fields confirmed from at least 5 different experiments.

### In Vitro multilineage Differentiation Studies

Adipogenesis, chondrogenesis and osteogenesis for h-ASCs were carried out in the appropriate induction media according to the manufacturer's protocol (Cambrex, MD http://www.cambrex.com). The differentiation phenotype was documented using oil red-O for adipocytes, Saffranin-O staining for chondrocytes and Alizarin staining for osteocytes. Dithizone (DTZ) (Sigma-Aldrich) stain of 10 mg/ml in DMSO (dimethyl sulphoxide) concentration used to stain islet like cell aggregates (ICAs).

### Total insulin content and release assays

For glucose stimulated insulin release assay, about 200–300 day-14 ICAs were handpicked in eppendorf tube. ICAs were then washed three times with PBS and incubated with freshly prepared KRBH buffer (120 mM NaCl, 5 mM Kcl, 2.5 mM CaCl_2_, 1.1 mM MgCl_2_, 25 mM NaHCO_3_, with 10 mM HEPES buffer and 0.1% BSA) without glucose for 3 to 6 h. ICAs were incubated with 100 µl KRBH buffer containing 5.5 mM glucose for 1 h at 37°C. The supernatant were collected and the same ICAs were further incubated 22 mM glucose for additional 1 h at 37°C. The human C-peptide concentrations were measured using (Ultra sensitive human C-peptide EIA kit, Mercodia, Uppsala, Sweden), mouse C-peptide concentrations were measured using mouse C-peptide I+II EIA kit (#YK013, Yanaihra Institute Inc., Japan, www.yanaihara.com). Total Intracellular C-peptide was extracted by incubating ICAs/h-ASCs overnight in acid ethanol (18 ml 10 M HCl/liter 70% ethanol) at 4°C. The total C-peptide content of ICAs was estimated after sonicating the ICA pellet in 200 µl acid/ethanol. The conversion factor for human C-peptide is 1 µg/l corresponds to 331 pmol/l (pM).

### In-vivo transplantation studies

Male Swiss albino mice aged 8–10 weeks were used for transplantation studies. The study was conducted adhering to the institution's guidelines for animal husbandry and has been approved by IAEC-NCCS/CPCSEA (Institutional animal ethical committee-NCCS/Committee for the Purpose of Control and Supervision of Experiments on Animals. Approval number: IACUC-Institutional Animal Care and Use Committee, EAF-Experimental Animal Facility/2006/B-56). Streptozotocin (STZ, Sigma-Aldrich) was injected intraperitoneally at 160 mg/kg of body weight, freshly dissolved in citrate buffer (pH 4.5). Blood glucose (BG) was measured by ACCU-CHEK (Roche, www.roche.com) from snipped tail. Only mice with BG levels stably above 200 mg/dl after the STZ injection were used for transplantation studies. For transplantation, around 1000–1200 day-10 ICAs were washed with PBS, suspended in 100 µl of sodium-alginate solution (1·2% w/v Alginic acid; Sigma-Chemical, USA, in 0·85% saline), packed into a biocompatible capsule (Polyurethane-Poly vinyl pyrrolidone-Interpenetrating network (PU-PVP-IPN), developed by SCTIMST, Kerala, India [23]. The ICAs-alginate capsule were sealed and dipped into 0·1 N acetic-acid and CaCl_2_ (0·15 M in distilled water) solution for gelling. These encapsulated ICAs were then implanted into the peritoneal cavity of diabetic mice. Fasting blood glucose levels were measured regularly using a glucometer after the mice were fasted for 6 h.

### Scanning Electron microscopy (SEM)

The differentiated ICAs were incubated in 2.5% phosphate buffer (0.1 M, pH 7.4) glutaraldehyde solution for 24 hrs. The tissue was post fixed in 1% OsO4 for 2 h at 4°C, dehydrated and dried. Specimens were then glued onto stubs, covered with gold in an S150 sputter coater and examined with a Hitachi S4000 field-emission (Hitachi Ltd. Tokyo, Japan) scanning electron microscope operating at 10 kV. (IIT, Mumbai).

### Statistical Analysis

Values are expressed as mean fold change ± S.E.M. from three different experiments unless otherwise indicated. Statistical analysis was done using Student's two tailed, t-test to determine the significance between different conditions. Prism4, Graphpad Software, San Diego, (http://www.graphpad.com) was used for all analysis.

## Supporting Information

Figure S1 **FACS based surface phenotypic characterization of h-ASCs.** h-ASCs clones at passage4 (n = 4), cells were harvested and labeled with antibodies against CD44-PE, CD13-PE, CD73-PE, CD90-PE, CD105-PE, CD59-PE, CD166-PE, CD38-PE, CD14-PE, CD71-PE, CD117-PE and Isotype control immunoglobulin-G PE and analyzed by FACS. Abbreviations: FACS, fluorescence-activated cell sorting; PE, phycoerythrin.
(TIF)

Figure S2 **Characterization of day 14 ICAs.** Immunostaining of day 14 ICAs for the expression of insulin and somatostatin (A), proliferation marker Ki-67 and insulin (B). The nuclei of the cells were stained with DAPI (4′, 6-diamidoino-2-phenylindole) (Scale bar = 20 µm).
(TIF)

Figure S3 **Expression of adipose tissue specific markers during the course of differentiation.** SYBR-Green based qRT-PCR analysis was carried out for day5 (Dif-d5) and day10 (Dif-d10) ICAs for adipose tissue specific markers like leptin, and adiponectin compared with undifferentiated h-ASCs (Undiff). Relative levels of gene expression were normalized to the GAPDH mRNA level(A). h-ASCs and day 14 ICAs are stained for the adipokine leptin (B). The nuclei of the cells were stained with DAPI (4′, 6-diamidoino-2-phenylindole) (Scale bar = 20 µm).
(TIF)

Figure S4 **Immunocytochemical characterization of 4-week in-vivo transplanted ICAs and h-ASCs.** Immunofluorescence staining of the retrieved ICAs after 28 days post-transplantation for the expression of C-peptide (Red) and insulin (green) (A). Encapsulated 4 week old transplanted h-ASCs also show c-peptide (red) and insulin (cyan) (B). The nuclei of the cells were stained blue with DAPI (4′, 6-diamidoino-2-phenylindole) (Scale bar = 20 µm).
(TIF)

Table S1 List of primers and antibodies used in this study.
(DOC)
