# Supplementary figures and images for: Islet-Like Cell Aggregates Generated from Human Adipose Tissue Derived Stem Cells Ameliorate Experimental Diabetes in Mice
Source: PLoS One. 2011 Jun 7;6(6):e20615. doi: 10.1371/journal.pone.0020615 (PMC3110196; doi:10.1371/journal.pone.0020615)

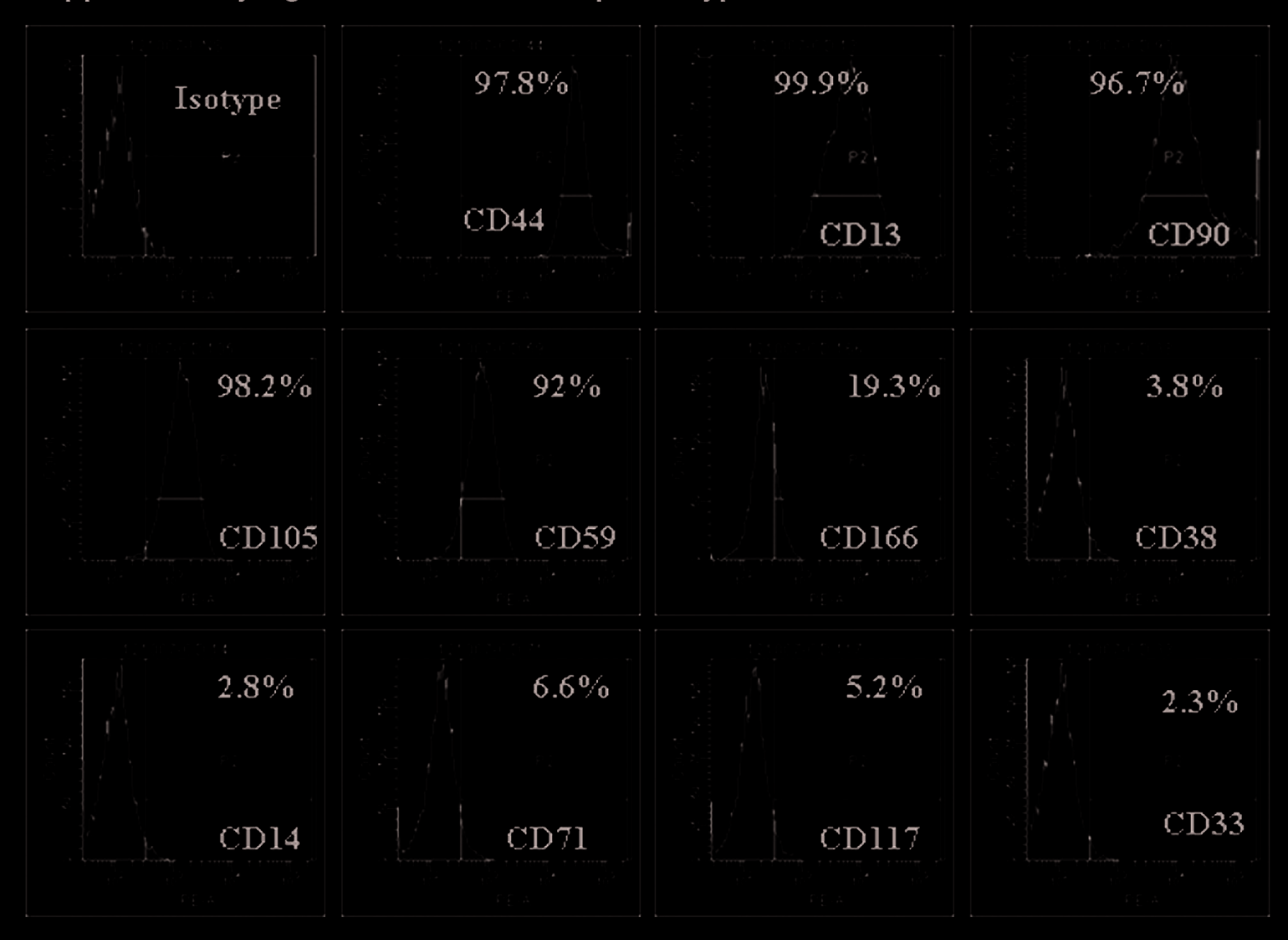

Supplement: Figure S1 — FACS based surface phenotypic characterization of h-ASCs. h-ASCs clones at passage4 (n = 4), cells were harvested and labeled with antibodies against CD44-PE, CD13-PE, CD73-PE, CD90-PE, CD105-PE, CD59-PE, CD166-PE, CD38-PE, CD14-PE, CD71-PE, CD117-PE and Isotype control immunoglobulin-G PE and analyzed by FACS. Abbreviations: FACS, fluorescence-activated cell sorting; PE, phycoerythrin. (TIF) [file pone.0020615.s001.tif]

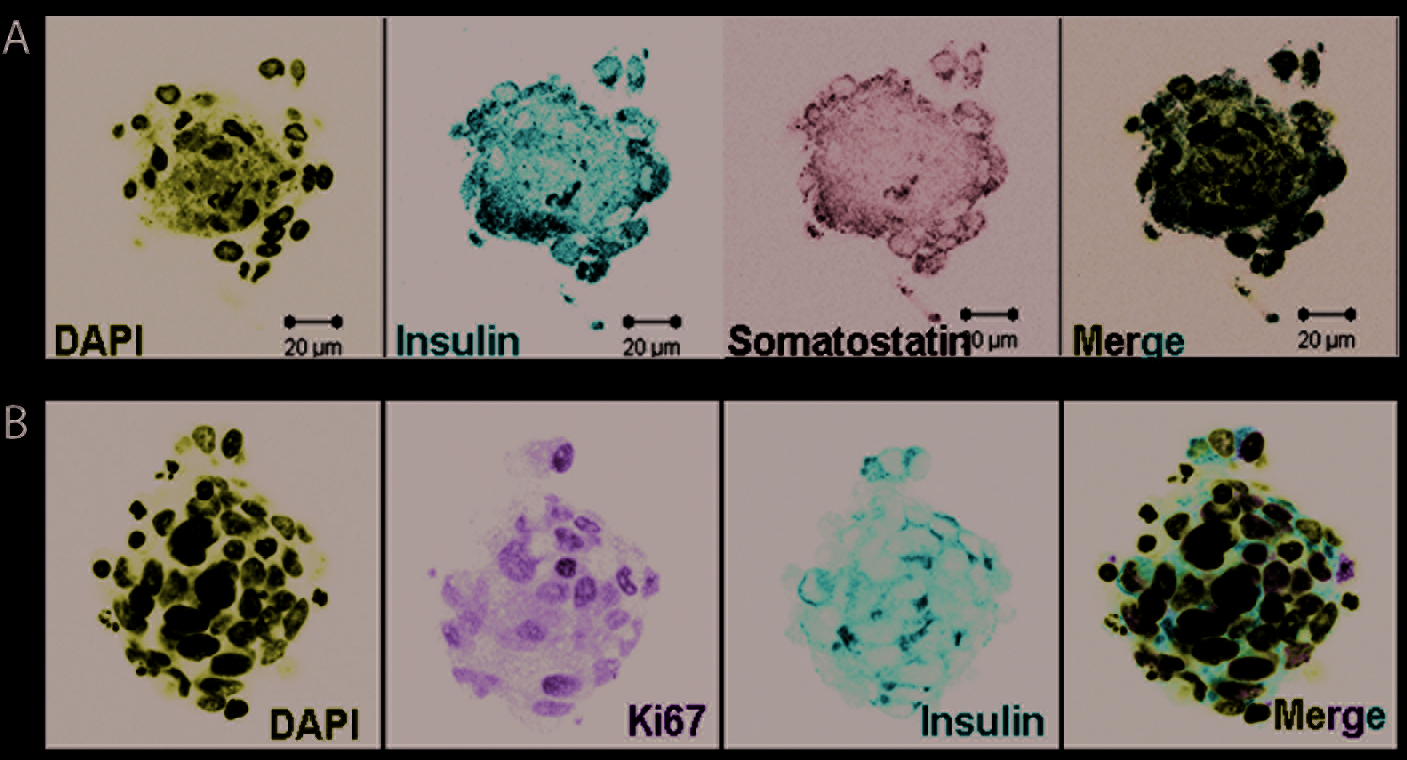

Supplement: Figure S2 — Characterization of day 14 ICAs. Immunostaining of day 14 ICAs for the expression of insulin and somatostatin (A), proliferation marker Ki-67 and insulin (B). The nuclei of the cells were stained with DAPI (4′, 6-diamidoino-2-phenylindole) (Scale bar = 20 µm). (TIF) [file pone.0020615.s002.tif]

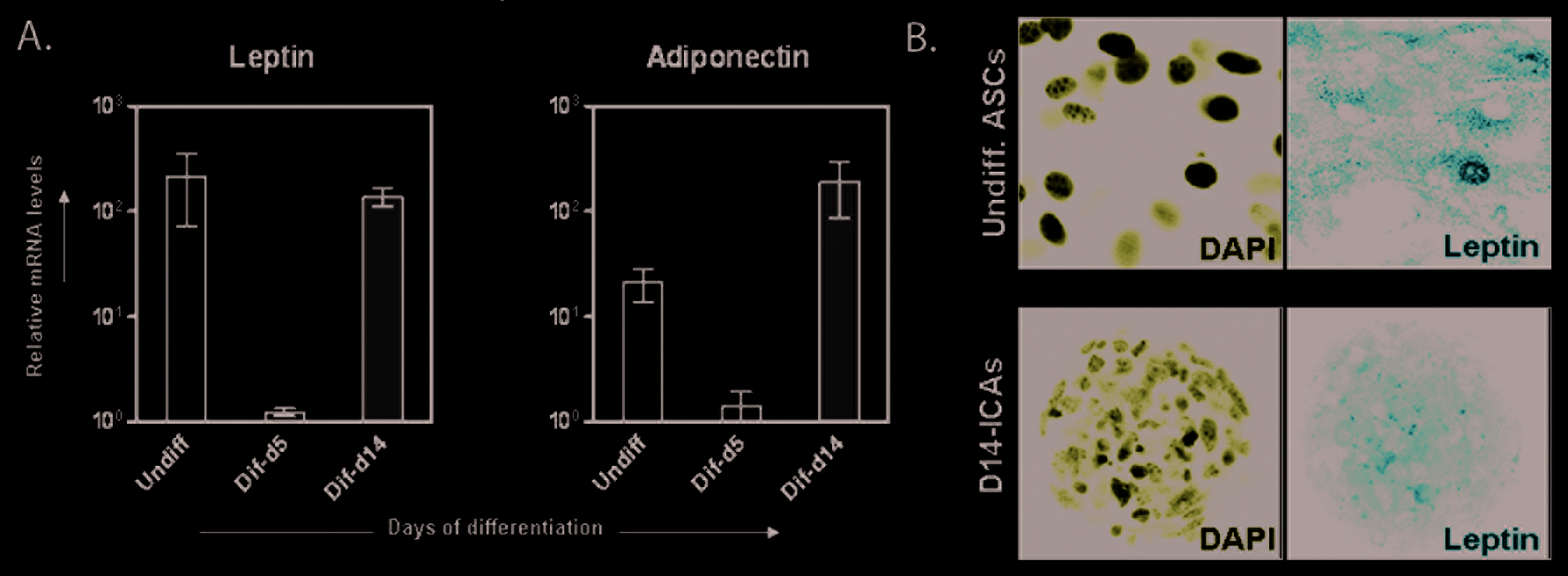

Supplement: Figure S3 — Expression of adipose tissue specific markers during the course of differentiation. SYBR-Green based qRT-PCR analysis was carried out for day5 (Dif-d5) and day10 (Dif-d10) ICAs for adipose tissue specific markers like leptin, and adiponectin compared with undifferentiated h-ASCs (Undiff). Relative levels of gene expression were normalized to the GAPDH mRNA level(A). h-ASCs and day 14 ICAs are stained for the adipokine leptin (B). The nuclei of the cells were stained with DAPI (4′, 6-diamidoino-2-phenylindole) (Scale bar = 20 µm). (TIF) [file pone.0020615.s003.tif]

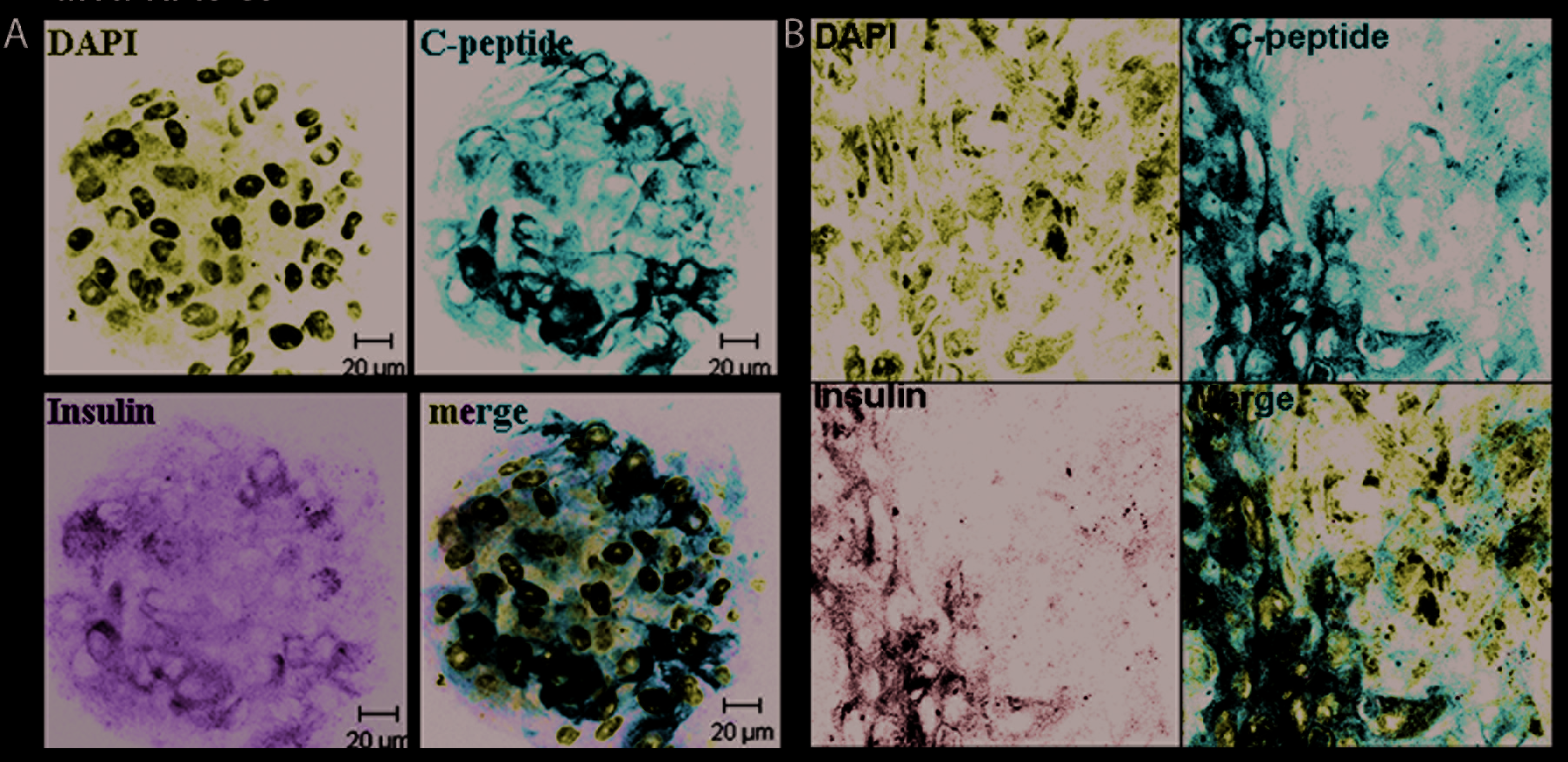

Supplement: Figure S4 — Immunocytochemical characterization of 4-week in-vivo transplanted ICAs and h-ASCs. Immunofluorescence staining of the retrieved ICAs after 28 days post-transplantation for the expression of C-peptide (Red) and insulin (green) (A). Encapsulated 4 week old transplanted h-ASCs also show c-peptide (red) and insulin (cyan) (B). The nuclei of the cells were stained blue with DAPI (4′, 6-diamidoino-2-phenylindole) (Scale bar = 20 µm). (TIF) [file pone.0020615.s004.tif]
