# Supplementary material for: Islet-Like Cell Aggregates Generated from Human Adipose Tissue Derived Stem Cells Ameliorate Experimental Diabetes in Mice
Source: PLoS One. 2011 Jun 7;6(6):e20615. doi: 10.1371/journal.pone.0020615 (PMC3110196; doi:10.1371/journal.pone.0020615)
Supplement: Table S1 — List of primers and antibodies used in this study. (DOC) [file pone.0020615.s005.doc]

**TABLE S1**

List of primers and antibodies used in this study

| S.N | **Gene** |  | Sequence 5'--3' | Product (bp) |
| --- | --- | --- | --- | --- |
|  |  |  |  |  |
| 1 | **H-GAPDH** |  | TTC GAC AGT CAG CCG CAT CTT CTT | 95 |
|  |  |  | ACC AAA TCC GTT GAC TCC GAC CTT |  |
| 2 | **H-Insulin** |  | AGA GGC CAT CAA GCA GAT CAC TGT | 125 |
|  |  |  | CAC AGG TGT TGG TTC ACA AAG GCT |  |
| 3 | **H-ghrelin** |  | AAG TGA TCG CCC ACA AGC CTT ACT | 99 |
|  |  |  | TGT ACA ACA GTC GTG GGA GTT GCT |  |
| 4 | **H-Glucagon** |  | TCT TGA TAA TCT TGC CGC CAG GGA | 186 |
|  |  |  | CAT GCA AAG CAA TGT GGC CTC AGA |  |
| 6 | **H-Somatostat** |  | TGA ACC CAA CCA GAC GGA GAA TGA | 151 |
|  |  |  | GAA ATT CTT GCA GCC AGC TTT GCG |  |
| 7 | **H-Pan-pol** |  | AAA GAC ACA AAG AGG ACA CGC TGG | 111 |
|  |  |  | TCG TAG GAG ACA GAA GGT GGC ATT |  |
| 8 | **H-Pdx-1** |  | TAC TGG ATT GGC GTT GTT TGT GGC | 133 |
|  |  |  | AGG GAG CCT TCC AAT GTG TAT GGT |  |
| 9 | **H-ngn3** |  | TAA GAG CGA GTT GGC ACT GAG CAA | 200 |
|  |  |  | TTT GAG TCA GCG CCC AGA TGT AGT |  |
| 10 | **H-Pax4** |  | AAT TCC CTG GAC TCA GGA CTG CTT | 120 |
|  |  |  | TTC CAA GCC ATA CAG TAG TGG GCA |  |
| 11 | **H-Pax6** |  | GCC CAG CTT CAC CAT GGC AAA TAA | 115 |
|  |  |  | ATC ATA ACT CCG CCC ATT CAC CGA |  |
| 12 | **H-NeuroD** |  | ATT GCA CCA GCC CTT CCT TTG ATG | 132 |
|  |  |  | TCG CTG CAG GAT AGT GCA TGG TAA |  |
| 13 | **H-Glut2** |  | AGC TGC ATT CAG CAA TTG GAC CTG | 128 |
|  |  |  | ATG TGA ACA GGG TAA AGG CCA GGA |  |
| 14 | **H-Nkx6.1** |  | AGA GAG TCA GGT CAA GGT CTG GTT | 103 |
|  |  |  | TGT CTC CGA GTC CTG CTT CTT CTT |  |
| 15 | **H-Nkx2.2** |  | GAC AAC TGG TGG CAG ATT TCG CTT | 114 |
|  |  |  | AGC CAC AAA GAA AGG AGT TGG ACC |  |
| 16 | **H-Hnf3B** |  | TTG CAG AGA CGC AAG GGA GAA GAA | 189 |
|  |  |  | ACA GTA GTG GAA ACC GGA GGC TTT |  |
| 17 | **H-isl-1** |  | TCT GTG GGC TGT TCA CCA ACT GTA | 128 |
|  |  |  | GCC GCA ACC AAC ACA TAG GGA AAT |  |
| 18 | **H-vimentin** |  | TGC AGG AGG CAG AAG AAT GGT ACA | 194 |
|  |  |  | TTC CAT TTC ACG CAT CTG GCG TTC |  |
| 19 | **H-nestin** |  | TGG CAA AGG AGC CTA CTC CAA GAA | 111 |
|  |  |  | ATC GGG ATT CAG CTG ACT TAG CCT |  |
| 20 | **H-Leptin** |  | TTG AAG CAA AGC ACC AGC TTC TCC | 147 |
|  |  |  | TCT TGC TCA GAT GAA CCC AAC CCT |  |
| 21 | **H-Adiponectin** |  | ATC CAA GGC AGG AAA GGA GAA CCT | 112 |
|  |  |  | TGG TAA AGC GAA TGG GCA TGT TGG |  |
|  |  |  |  |  |
| 22 | **H-CK-19** |  | ATA TGA GGT CAT GGC CGA GCA GAA | 170 |
|  |  |  | ACT GCA GCT CAA TCT CAA GAC CCT |  |
| 23 | **H-Sox17** |  | AGG AAA TCC TCA GAC TCC TGG GTT | 111 |
|  |  |  | CCC AAA CTG TTC AAG TGG CAG ACA |  |
| 24 | **H-Gata4** |  | TAC ATC AGC TTC CGG AAC CAC CAA | 87 |
|  |  |  | AAT CCA GCA TTG AGC AAA GGG CTC |  |

| **SN** | **Antibody** | **Company** | **Dilution** |
| --- | --- | --- | --- |
| 1 | **Nestin** | Millipore, www.millipore.com | 1-100 |
| 2 | **Vimentin** | Sigma , www.sigma-aldrich.com | 1-100 |
| 3 | **α-SMA** | Sigma, www.sigma-aldrich.com | 1-100 |
| 4 | **Fibronectin** | Millipore, www.millipore.com | 1-100 |
| 5 | **Ki-67** | Millipore, www.millipore.com | 1-150 |
| 6 | **C-peptide** | Millipore, www.millipore.com | 1-100 |
| 7 | **Pdx-1** | Millipore, www.millipore.com | 1-100 |
| 8 | **Insulin** | LINCO, www.millipore.com | 1-200 |
| 9 | **Glucagon** | Sigma, www.sigma-aldrich.com | 1-200 |
| 10 | **Somatostatin** | Dako, www.dako.com | 1-200 |
| 11 | **Glut2** | Millipore, www.millipore.com | 1-100 |
| 12 | **CD73-PE** | BD, www.bdbiosciences.com | 1-100 |
| 13 | **CD90-APC** | BD, www.bdbiosciences.com | 1-100 |
| 14 | **CD105-PE** | Santa Cruz biotechnology inc, | 1-100 |
| 15 | **CD29-FITC** | BD, www.bdbiosciences.com | 1-100 |
| 16 | **CD44-PE** | BD, www.bdbiosciences.com | 1-100 |
